# Supplementary material for: Modeling glioblastoma heterogeneity as a dynamic network of cell states
Source: Mol Syst Biol. 2021 Sep 16;17(9):e10105. doi: 10.15252/msb.202010105 (PMC8444284; doi:10.15252/msb.202010105)
Supplement: Supplementary file 5 — Source Data for Figure 3 [file MSB-17-e10105-s001.zip › Figure3A_sourcedata/GSEA_3065/nosigdb_state1.GseaPreranked.1622718409535/TISIG_PMID_32649888.html]

Details for gene set TISIG\_PMID\_32649888[GSEA]

|  || Dataset | state1 |
| Phenotype | NoPhenotypeAvailable |
| Upregulated in class | na\_neg |
| GeneSet | TISIG\_PMID\_32649888 |
| Enrichment Score (ES) | -0.778952 |
| Normalized Enrichment Score (NES) | -2.276664 |
| Nominal p-value | 0.0 |
| FDR q-value | 0.0 |
| FWER p-Value | 0.0 |
Table: GSEA Results Summary

  

Fig 1: Enrichment plot: TISIG\_PMID\_32649888      
 Profile of the Running ES Score & Positions of GeneSet Members on the Rank Ordered List

  

| PROBE | GENE SYMBOL | GENE\_TITLE | RANK IN GENE LIST | RANK METRIC SCORE | RUNNING ES | CORE ENRICHMENT || 1 | KCNIP1 |  |  | 1126 | 0.119 | -0.0993 | No |
| 2 | CKB |  |  | 1368 | 0.098 | -0.1114 | No |
| 3 | DIRAS2 |  |  | 1434 | 0.093 | -0.1062 | No |
| 4 | EGFR |  |  | 1474 | 0.091 | -0.0987 | No |
| 5 | LRRC17 |  |  | 2509 | 0.039 | -0.1987 | No |
| 6 | RASSF2 |  |  | 3195 | 0.020 | -0.2657 | No |
| 7 | KLHL4 |  |  | 3939 | 0.005 | -0.3405 | No |
| 8 | ZIC1 |  |  | 4766 | -0.008 | -0.4234 | No |
| 9 | KCNJ16 |  |  | 6368 | -0.036 | -0.5814 | No |
| 10 | SPRY4 |  |  | 6504 | -0.039 | -0.5903 | No |
| 11 | GAS1 |  |  | 6510 | -0.039 | -0.5859 | No |
| 12 | ARC |  |  | 6612 | -0.041 | -0.5909 | No |
| 13 | HOPX |  |  | 7850 | -0.075 | -0.7071 | No |
| 14 | LRP4 |  |  | 8181 | -0.089 | -0.7294 | No |
| 15 | NDRG4 |  |  | 8182 | -0.089 | -0.7182 | No |
| 16 | CDH4 |  |  | 8271 | -0.093 | -0.7153 | No |
| 17 | TRIB2 |  |  | 8853 | -0.129 | -0.7580 | No |
| 18 | MEOX2 |  |  | 9060 | -0.148 | -0.7603 | Yes |
| 19 | POU3F2 |  |  | 9173 | -0.162 | -0.7512 | Yes |
| 20 | ATP1B2 |  |  | 9441 | -0.210 | -0.7518 | Yes |
| 21 | DPP6 |  |  | 9606 | -0.262 | -0.7353 | Yes |
| 22 | FGFR3 |  |  | 9621 | -0.266 | -0.7031 | Yes |
| 23 | NES |  |  | 9626 | -0.270 | -0.6694 | Yes |
| 24 | CSPG5 |  |  | 9721 | -0.330 | -0.6372 | Yes |
| 25 | MLC1 |  |  | 9728 | -0.338 | -0.5950 | Yes |
| 26 | SOX9 |  |  | 9754 | -0.358 | -0.5523 | Yes |
| 27 | SDC3 |  |  | 9756 | -0.360 | -0.5068 | Yes |
| 28 | WSCD1 |  |  | 9808 | -0.448 | -0.4553 | Yes |
| 29 | C1orf61 |  |  | 9813 | -0.479 | -0.3952 | Yes |
| 30 | SLC1A3 |  |  | 9823 | -0.509 | -0.3317 | Yes |
| 31 | EDNRB |  |  | 9824 | -0.509 | -0.2673 | Yes |
| 32 | FABP7 |  |  | 9825 | -0.513 | -0.2025 | Yes |
| 33 | CCND2 |  |  | 9842 | -0.611 | -0.1270 | Yes |
| 34 | TTYH1 |  |  | 9872 | -1.033 | 0.0007 | Yes |
Table: GSEA details [plain text format]

  

Fig 2: TISIG\_PMID\_32649888: Random ES distribution      
 Gene set null distribution of ES for **TISIG\_PMID\_32649888**
